# Supplementary material for: Can Milk Affect Recovery from Simulated Team-Sport Match Play?
Source: Nutrients. 2019 Dec 31;12(1):112. doi: 10.3390/nu12010112 (PMC7019310; doi:10.3390/nu12010112)
Supplement: Supplementary file 1 [file nutrients-12-00112-s001.pdf]

Table S1. Within-group effects over time for dependent variables.

Variable	Timeframe	Mean effect [a], ±90% CI or Factor effect x/÷ 90% CI	Qualitative inference (ES)[c]
**Peak Torque 60°/s Extension**			
MILK	B-24	-6.1, ±3.6	Small (0.33) decrease **
	B-48	-4.7, ±3.4	Small (0.26) decrease*
	B-72	-4.5, ±4.7	Small (0.25) decrease*
	B-96	-3.3, ±4.7	Trivial decrease*
	48-72	0.2, ±5.6	Trivial**
	48-96	1.4, ±5.5	Trivial*
CHO	B-24	-10.2, ±2.9	Moderate (0.98) decrease****
	B-48	-9.6, ±5.0	Moderate (0.93) decrease***
	B-72	-6.8, ±4.2	Moderate (0.65) decrease***
	B-96	-3.6, ±3.2	Small (0.35) decrease**
	48-72	3.0, ±2.3	Small (0.28) increase**
	48-96	6.7, ±4.9	Small (0.58) increase***
**Peak Torque 60°/s Flexion**			
MILK	B-24	-5.0, ±6.1	Small (0.41) decrease**
	B-48	-5.4, ±5.5	Small (0.44) decrease**
	B-72	-3.0, ±5.1	Small (0.26) decrease*
	B-96	-3.2, ±5.6	Unclear
	48-72	2.5, ±6.1	Unclear
	48-96	2.3, ±3.8	Unclear
CHO	B-24	-11.6, ±4.9	Moderate (0.73) decrease***
	B-48	-15.3, ±9.5	Moderate (0.90) decrease***
	B-72	-12.3, ±11.5	Moderate (0.67) decrease**
	B-96	-5.4, ±9.7	Unclear
	48-72	3.6, ±3.4	Small (0.23) increase*
	48-96	11.7, ±3.1	Moderate (0.62) increase****
**Peak Torque 180°/s Extension**			
MILK	B-24	-4.0, ±4.6	Small (0.27) decrease*
	B-48	-1.7, ±5.0	Trivial**
	B-72	-1.6, ±5.3	Trivial**
	B-96	-1.8, ±5.9	Trivial decrease*
	48-72	0.2, ±2.9	Trivial***
	48-96	-0.1, ±4.0	Trivial**

CHO	B-24	-3.2, ±2.9	Small (0.27) decrease*
	B-48	-3.4, ±4.7	Small (0.28) decrease*
	B-72	-4.6, ±4.4	Small (0.38) decrease**
	B-96	-1.5, ±3.1	Trivial decrease*
	48-72	-1.2, ±3.7	Trivial decrease*
	48-96	2.0, ±3.5	Unclear
**Peak Torque 180°/s Flexion**			
MILK	B-24	-4.7, ±5.0	Small(0.25) decrease*
	B-48	-3.1, ±1.8	Small (0.20) decrease*
	B-72	-4.2, ±3.4	Small (0.20) decrease*
	B-96	-4.5, ±5.9	Small (0.21) decrease*
	48-72	-1.1, ±4.4	Trivial**
	48-96	-1.5, ±5.9	Unclear
CHO	B-24	-7.0, ±5.2	Small (0.39) decrease**
	B-48	-9.1, ±3.7	Small (0.47) decrease***
	B-72	-6.1, ±7.6	Small (0.32) decrease*
	B-96	-5.3, ±7.3	Small (0.28) decrease*
	48-72	3.3, ±6.1	Unclear
	48-96	4.2, ±6.5	Unclear
**CMJ**			
MILK	B-24	-6.1, ±2.1	Small (0.39) decrease***
	B-48	-4.7, ±3.6	Small (0.30) decrease**
	B-72	-4.7, ±2.8	Small (0.29) decrease**
	B-96	-3.3, ±3.5	Trivial decrease*
	48-72	0.1, ±3.7	Trivial**
	48-96	1.5, ±1.4	Unclear
CHO	B-24	-7.5, ±5.7	Moderate (0.60) decrease**
	B-48	-8.6, ±7.3	Moderate (0.66) decrease**
	B-72	-6.5, ±5.5	Small (0.51) decrease**
	B-96	-5.3, ±7.0	Small (0.41) decrease**
	48-72	2.4, ±4.0	Unclear
	48-96	3.6, ±3.7	Small (0.25) increase**
**RSI**			
MILK	B-24	-8.5, ±6.1	Small (0.39) decrease**
	B-48	-13.5, ±5.2	Moderate (0.69) decrease****
	B-72	-17.8, ±5.4	Moderate (0.92) decrease****
	B-96	-10.0, ±6.0	Small (0.49) decrease***
	48-72	-5.0, ±6.4	Small (0.23) decrease*
	48-96	4.1, ±6.7	Unclear
CHO	B-24	-15.0, ±6.4	Moderate (0.86) decrease***
	B-48	-14.8, ±5.6	Moderate (0.87) decrease****

	B-72	-10.8, ±10.7	Small (0.59) decrease**
	B-96	-2.8, ±12.2	Trivial decrease*
	48-72	4.9, ±7.8	Unclear
	48-96	14.1, ±10.0	Moderate (0.74) increase***
**5m sprint**			
MILK	B-24	1.3, ±1.5	Trivial decrease*
	B-48	1.5, ±2.3	Small (0.22) decrease*
	B-72	2.2, ±2.6	Small (0.33) decrease**
	B-96	-0.3, ±4.5	Unclear
	48-72	0.7, ±1.9	Trivial*
	48-96	-1.7, ±3.5	Unclear
CHO	B-24	3.1, ±2.1	Moderate (0.76) decrease***
	B-48	3.8, ±2.7	Moderate (0.95) decrease***
	B-72	4.8, ±3.2	Large (1.23) decrease***
	B-96	2.8, ±3.0	Moderate (0.70) decrease**
	48-72	1.0, ±1.5	Small (0.27) decrease*
	48-96	-1.0, ±1.5	Unclear
**10m sprint**			
MILK	B-24	2.2, ±1.4	Small (0.40) decrease**
	B-48	1.2, ±1.8	Small (0.21) decrease*
	B-72	1.9, ±1.7	Small (0.34) decrease**
	B-96	-0.8, ±0.8	Trivial increase*
	48-72	0.7, ±1.4	Trivial decrease*
	48-96	-1.9, ±1.5	Small (0.36) increase**
CHO	B-24	2.2, ±1.9	Moderate (0.61) decrease**
	B-48	2.8, ±2.0	Moderate (0.77) decrease***
	B-72	2.5, ±2.1	Moderate (0.71) decrease**
	B-96	1.1, ±1.4	Small (0.30) decrease*
	48-72	-0.2, ±1.4	Unclear
	48-96	-1.6, ±1.9	Small (0.47) increase**
**20m sprint**			
MILK	B-24	2.6, ±1.7	Small (0.40) decrease**
	B-48	2.1, ±1.3	Small (0.33) decrease**
	B-72	2.7, ±1.7	Small (0.43) decrease**
	B-96	0.2, ±3.3	Unclear
	48-72	0.6, ±1.2	Trivial*
	48-96	-2.2, ±3.1	Unclear
CHO	B-24	2.0, ±1.1	Small (0.36) decrease**
	B-48	2.0, ±2.1	Small (0.38) decrease**
	B-72	2.4, ±1.3	Small (0.45) decrease***
	B-96	1.2, ±1.1	Small (0.22) decrease*
	48-72	0.4, ±1.8	Trivial decrease*
	48-96	-0.8, 2.2	Unclear

**RFD**			
MILK	B-24	-12.1, ±17.4	Small (0.35) decrease*
	B-48	-21.6, ±11.6	Moderate (0.68) decrease***
	B-72	-14.6, ±13.7	Small (0.45) decrease**
	B-96	-8.2, ±9.0	Trivial decrease*
	48-72	8.9, ±14.6	Unclear
	48-96	17.1, 20.4	Small (0.49) increase*
CHO	B-24	-35.5, ±18.3	Moderate (0.76) decrease***
	B-48	-30.4, ±14.0	Moderate (0.71) decrease***
	B-72	-26.7, ±14.9	Moderate (0.87) decrease***
	B-96	-33.2, ±22.3	Moderate (0.77) decrease**
	48-72	-3.1, ±25.4	Trivial decrease*
	48-96	-4.0, ±23.6	Trivial decrease*
**Creatine Kinase**			
MILK	B-2	1.7 x/÷ 1.4	Moderate (0.82) increase***
	B-24	1.7 x/÷ 1.3	Moderate (0.77) increase***
	B-48	1.1 x/÷1.3	Trivial increase*
	B-52	2.3 x/÷1.2	Large (1.86) increase****
	B-72	1.7 x/÷ 1.7	Moderate (1.08) increase**
	B-96	1.2 x/÷1.3	Small 0.20) increase*
	48-52	2.3 x/÷ 1.2	Large (1.86) increase***
	48-72	1.6 x/÷ 1.7	Moderate (1.11) increase**
	48-96	1.2 x/÷ 1.2	Small (0.23) increase*
CHO	B-2	1.9 x/÷ 1.4	Large (1.37) increase***
	B-24	1.7 x/÷ 1.7	Large (1.79) increase**
	B-48	1.3 x/÷ 1.5	Small (0.55) increase**
	B-52	2.3 x/÷ 1.2	Large (1.82) increase****
	B-72	1.7 x/÷ 1.6	Moderate (1.14) increase**
	B-96	1.2 x/÷ 1.4	Small (0.35) increase*
	48-52	1.8 x/÷ 1.2	Large (1.37) increase****
	48-72	1.3 x/÷ 1.4	Small (0.59) increase **
	48-96	0.9 x/÷ 1.3	Unclear
**hsCRP**			
MILK	B-2	1.1 x/÷ 1.4	Unclear
	B-24	1.5 x/÷ 1.5	Small (0.46) increase**
	B-48	1.2 x/÷ 1.6	Unclear
	B-52	1.1 x/÷ 1.3	Unclear
	B-72	1.4 x/÷1.6	Unclear
	B-96	1.1 x/÷ 1.8	Unclear
	48-52	1.0 x/÷ 1.1	Trivial**
	48-72	1.1 x/÷ 1.2	Trivial increase*
	48-96	0.9 x/÷ 1.4	Unclear
CHO	B-2	0.9 x/÷ 1.2	Small (0.31) increase*

	B-24	0.9 x/÷ 1.4	Unclear
	B-48	0.7 x/÷ 1.4	Small (0.47) increase**
	B-52	1.1 x/÷1.3	Unclear
	B-72	1.3 x/÷ 1.3	Unclear
	B-96	1.2 x/÷ 1.7	Unclear
	48-52	1.6 x/÷ 2.0	Very large (2.05) increase**
	48-72	1.8 x/÷ 2.1	Very large (2.74) increase**
	48-96	1.6 x/÷ 2.0	Large (1.72) increase**
**Soreness (isokinetic)**			
MILK	B-24	5.1, ±0.4	Large increase****
	B-48	6.0, ±0.6	Large increase****
	B-72	6.1, ±0.9	Large increase****
	B-96	4.4, ±1.3	Moderate increase****
	48-72	0.1, ±0.6	Unclear
	48-96	-1.6, ±1.2	Small decrease**
CHO	B-2	5.8, ±0.7	Large increase****
	B-48	6.1, ±0.9	Large increase****
	B-72	6.6, ±0.8	Large increase****
	B-96	5.2, ±1.0	Large increase****
	48-72	0.5, ±0.8	Trivial increase**
	48-96	-0.9, ±0.9	Trivial decrease**
**Soreness (squat)**			
MILK	B-24	3.3, ±1.4	Moderate increase***
	B-48	4.2, ±1.1	Moderate increase****
	B-72	4.4, ±1.4	Moderate increase****
	B-96	3.2, ±1.1	Moderate increase****
	48-72	0.2, ±1.1	Unclear
	48-06	-1.1, ±0.9	Small decrease**
CHO	B-24	3.4, ±1.2	Moderate increase****
	B-48	5.3, ±0.7	Large increase****
	B-72	5.0, ±1.0	Large increase****
	B-96	3.6, ±1.1	Moderate increase****
	48-72	-0.3, ±1.3	Unclear
	48-96	-1.7, ±1.2	Small decrease***
**Tiredness**			
MILK	B-24	4.2, ±1.2	Moderate increase****
	B-48	5.0, ±1.3	Large increase****
	B-72	4.9, ±1.0	Moderate increase****
	B-96	3.8, ±0.9	Moderate increase****
	48-72	-0.1, ±0.9	Unclear
	48-96	-1.2, ±1.1	Trivial decrease**
CHO	B-24	4.3, ±0.8	Moderate increase****

	B-48	6.0, ±1.0	Large increase****
	B-72	6.0, ±1.1	Large increase****
	B-96	4.3, ±1.1	Moderate increase****
	48-72	0.0, ±1.2	Unclear
	48-96	-1.2, ±1.1	Small decrease***
**DALDA Part B**			
MILK	B-24	2.9, ±1.3	Small increase***
	B-48	4.3, ±2.1	Small increase***
	B-72	4.2, ±2.3	Small increase***
	B-96	3.8, ±2.9	Small increase***
	48-72	-0.1, ±1.2	Unclear
	48-96	-0.5, ±1.6	Unclear
CHO	B-24	5.3, ±1.6	Small increase****
	B-48	8.0, ±1.7	Moderate increase****
	B-72	7.4, ±2.1	Moderate increase****
	B-96	5.8, ±2.4	Small increase****
	48-72	-0.6, ±1.0	Trivial decrease**
	48-96	-2.2, ±2.0	Trivial decrease**
**LOOH**			
MILK	B-2	1.0 x/÷ 1.4	Unclear
	B-24	1.0 x/÷ 1.1	Trivial**
	B-48	1.0 x/÷ 1.2	Trivial*
	B-52	0.9 x/÷ 1.2	Unclear
	B-72	1.0 x/÷ 1.3	Unclear
	B-96	1.0 x/÷ 1.3	Unclear
	48-52	1.0 x/÷ 1.1	Trivial**
	48-72	1.0 x/÷ 1.0	Trivial increase*
	48-96	1.0 x/÷ 1.2	Trivial increase*
CHO	B-2	1.1 x/÷ 1.1	Trivial increase*
	B-24	0.9 x/÷ 1.1	Small (0.33) decrease*
	B-48	1.0 x/÷ 1.2	Unclear
	B-52	1.0 x/÷ 1.2	Unclear
	B-72	1.0 x/÷ 1.2	Trivial*
	B-96	0.8 x/÷1.3	Unclear
	48-52	1.0 x/÷ 1.3	Trivial*
	48-72	1.0 x/÷ 1.3	Unclear
	48-96	0.9 x/÷ 1.4	Trivial decrease*
**PC**			
MILK	B-2	1.0 x/÷ 1.8	Unclear
	B-24	1.1 x/÷ 1.5	Trivial*
	B-48	1.0 x/÷ 1.3	Unclear
	B-52	1.0 x/÷ 1.5	Unclear
	B-72	0.8 x/÷ 1.7	Unclear

	B-96	1.0 x/÷ 1.4	Unclear
	48-52	0.9 x/÷ 1.7	Unclear
	48-72	0.8 x/÷ 2.0	Unclear
	48-96	1.0 x/÷ 1.5	Unclear
CHO	B-2	0.9 x/÷ 1.5	Unclear
	B-24	1.0 x/÷ 1.2	Unclear
	B-48	1.0 x/÷ 1.4	Unclear
	B-52	0.9 x/÷ 1.2	Unclear
	B-72	0.9 x/÷ 1.2	Unclear
	B-96	1.0 x/÷ 1.4	Unclear
	48-52	0.9 x/÷ 1.5	Unclear
	48-72	0.9 x/÷ 1.5	Unclear
	48-96	1.0 x/÷ 1.4	Unclear

[a] Mean effect refers to Baseline minus 24h/48h/72h/96h; [b] ± 90% CI: add and subtract this number to the mean effect to obtain the 90% confidence intervals for the true difference; [c]Qualitative Inference represents the likelihood that the true value will have the observed magnitude *Possible,**Likely, ***Very likely, **** Most likely.

Table S2. Within-group effects from STG1 to STG2.

Variable	Mean effect[a], ±90% CI[b]	Qualitative inference (ES)[c]
**5m sprint performance**		
MILK	3.0, ±1.7	Small (0.31) decrease*
CHO	2.3, ±7.4	Small (0.36) decrease***
**15m sprint performance**		
MILK	-3.3, ±2.5	Small (0.38) increase**
CHO	-5.9, ±7.2	Moderate (0.74) increase**
**CMJ**		
MILK	-1.0, ±4.0	Trivial**
CHO	-0.4, ±2.0	Trivial**
**Lap time**		
MILK	-0.5, ±3.5	Unclear
CHO	0.1, ±2.1	Trivial**
**Heart rate**		
MILK	0.9, ±1.0	Trivial**
CHO	0.6, ±0.9	Trivial**
**RPE**		
MILK	0.0, ±0.8	Trivial*
CHO	-0.8, ±0.7	Trivial decrease**

[a] Mean effect refers to STG1 minus STG2; [b] ± 90% CI: add and subtract this number to the mean effect to obtain the 90% confidence intervals for the true difference; [c]Qualitative Inference represents the likelihood that the true value will have the observed magnitude, *Possible, **Likely, ***Very likely.
